# Serum and macular response to carotenoid-enriched egg supplementation in human subjects: the Egg Xanthophyll Intervention clinical Trial (EXIT)

**DOI:** 10.1017/S0007114516003895

**Published:** 2017-01-14

**Authors:** David Kelly, John M. Nolan, Alan N. Howard, Jim Stack, Kwadwo O. Akuffo, Rachel Moran, David I. Thurnham, Jessica Dennison, Katherine A. Meagher, Stephen Beatty

**Affiliations:** 1 Macular Pigment Research Group, Nutrition Research Centre Ireland, School of Health Sciences, Waterford Institute of Technology, Waterford X91 K236, Republic of Ireland; 2 Howard Foundation, Cambridge CB25 ONW, UK; 3 Downing College, University of Cambridge, Cambridge CB2 1DQ, UK; 4 Northern Ireland Centre for Food and Health (NICHE), University of Ulster, Coleraine BT52 1SA, UK

**Keywords:** Lutein, Zeaxanthin, *Meso*-zeaxanthin, Macular pigment, Carotenoid-enriched eggs, Serum carotenoids, Cholesterol

## Abstract

The macular carotenoids lutein (L), zeaxanthin (Z) and *meso*-zeaxanthin (MZ) accumulate at the macula, where they are collectively referred to as macular pigment (MP). Augmentation of this pigment, typically achieved through diet and supplementation, enhances visual function and protects against progression of age-related macular degeneration. However, it is known that eggs are a rich dietary source of L and Z, in a highly bioavailable matrix. In this single-blind placebo-controlled study, L- and MZ-enriched eggs and control non-enriched eggs were fed to human subjects (mean age 41 and 35 years, respectively) over an 8-week period, and outcome measures included MP, visual function and serum concentrations of carotenoids and cholesterol. Serum carotenoid concentrations increased significantly in control and enriched egg groups, but to a significantly greater extent in the enriched egg group (*P*<0·001 for L, Z and MZ). There was no significant increase in MP in either study group post intervention, and we saw no significant improvement in visual performance in either group. Total cholesterol increased significantly in each group, but it did not exceed the upper limit of the normative range (6·5 mmol/l). Therefore, carotenoid-enriched eggs may represent an effective dietary source of L, Z and MZ, reflected in significantly raised serum concentrations of these carotenoids, and consequentially improved bioavailability for capture by target tissues. However, benefits in terms of MP augmentation and /or improved visual performance were not realised over the 8-week study period, and a study of greater duration will be required to address these questions.

Lutein (L), zeaxanthin (Z) and *meso*-zeaxanthin (MZ) are oxygenated xanthophylls belonging to a group of plant pigments known as carotenoids^(^ ^1^ ^)^. These three nutrients accumulate at the back of the human eye, in the central part of the retina (the macula, a specialised tissue that mediates central vision)^(^ ^2^ ^)^, where they are collectively known as macular pigment (MP). MP has been shown to protect against progression of age-related macular degeneration (AMD), a disease of the macula, which is the leading cause of age-related blindness in the developed world^(^ ^3^ ^–^ ^5^ ^)^. This protection conferred upon the macula is achieved through MP’s antioxidant properties, which enable it to quench unstable reactive oxygen species and prevent consequential damage to the retinal photoreceptors^(^ ^6^ ^–^ ^9^ ^)^, and also through its optical light-filtering properties, which facilitate absorption of high-energy, short-wavelength damaging blue light^(^ ^10^ ^,^ ^11^ ^)^. MP has also been shown to improve visual function^(^ ^12^ ^)^ in both diseased^(^ ^13^ ^–^ ^19^ ^)^ and non-diseased (healthy)^(^ ^20^ ^)^ eyes. We know that in healthy subjects, free of retinal disease (similar to the subjects recruited into this trial), enrichment of MP following supplementation with a combination of L, Z and MZ exhibits clinically meaningful improvements in visual function^(^ ^21^ ^)^. In addition, other *in vivo* murine work has shown that L has the capacity to both inhibit downstream pathological signals of oxidative stress in the retina and preserve visual function at the molecular level^(^ ^22^ ^)^. Moreover, in similar *in vivo* work, L was shown to be beneficial in tight-junction repair in the retina^(^ ^23^ ^)^, whereas human supplementation trials have indicated that L may positively alter the alternative complement activation pathway by lowering systemic levels of factor D, which has been found in elevated levels in the blood of AMD patients^(^ ^17^ ^)^.

Recent studies have also confirmed the presence of L and Z in the non-human primate brain^(^ ^24^ ^)^ and the human brain^(^ ^25^ ^–^ ^27^ ^)^, and in concentrations that are proportional to retinal concentrations of these carotenoids. Interestingly, there is a growing body of evidence that these carotenoids may be important in maintaining optimal cognitive function^(^ ^28^ ^–^ ^31^ ^)^.

To date, the majority of studies investigating the role of these carotenoids for vision and cognitive function have relied on the use of commercially available supplement formulations. Recently, it has been suggested that novel nutrient-enriched (functional) foods may offer an alternative and a possibly more convenient source of nutrients to consumers, with eggs and milk being two potential candidates for L, Z and MZ^(^ ^32^ ^–^ ^37^ ^)^.

Daily intake of L and Z in a typical Western diet is 1–3 mg^(^ ^38^ ^)^, with up to 78 % sourced from vegetable intake, such as spinach and kale, and maize products^(^ ^39^ ^,^ ^40^ ^)^. In contrast, MZ has only been identified (in trace amounts) in seafood such as trout, sardines, salmon, shrimp and turtles^(^ ^41^ ^,^ ^42^ ^)^. Interestingly, and in spite of the lack of dietary MZ, this xanthophyll still accounts for one-third of total MP^(^ ^43^ ^)^, and studies have suggested that MZ is produced by isomerisation of L in the macula^(^ ^44^ ^)^, but this proposed process is poorly understood^(^ ^45^ ^)^. Importantly, MZ has been shown to be MP’s centrally dominant constituent carotenoid^(^ ^46^ ^)^. In addition, it has been shown (*in vitro*) that MZ exhibits the greatest antioxidant activity of the three carotenoids, but the combination of all three (L, Z and MZ) exhibits an even greater antioxidant activity^(^ ^47^ ^)^.

The bioavailability of carotenoids in the diet is determined by the characteristics of the food matrix in which they are delivered and by possible interactions with other dietary components^(^ ^48^ ^)^. For example, localisation of L and Z within the chromoplasts of vegetables reduces their bioavailability to serum when ingested and, accordingly, decreasing the food particle size (by chopping, blending, pureeing, etc.) and breaking the cell wall (by cooking), before consumption, is often necessary for optimal absorption^(^ ^49^ ^–^ ^53^ ^)^. Of interest, studies have shown that the bioavailability of the macular carotenoids may be enhanced when dissolved in a lipid matrix, such as that of the egg yolk, which contains digestible lipids such as cholesterol, TAG and phospholipids, as this facilitates efficient digestion and absorption^(^ ^54^ ^)^. Interestingly, several studies have shown that the bioavailability of L and Z from eggs is superior to that from other food sources and from dietary supplements^(^ ^38^ ^,^ ^48^ ^,^ ^55^ ^,^ ^56^ ^)^. This is likely because of the presence of HDL found in high concentrations in eggs, which is known to be the primary transport vehicle for L in the bloodstream^(^ ^57^ ^–^ ^59^ ^)^.

Hen eggs are produced on an industrial scale, and are consumed as part of a typical diet, and they provide many nutritional benefits^(^ ^60^ ^)^. It has been well documented that supplementation with MP’s constituent carotenoids increases both the serum and retinal concentrations of these nutrients^(^ ^1^ ^,^ ^14^ ^,^ ^20^ ^,^ ^61^ ^)^, and this has also been demonstrated using carotenoid-rich foods, including eggs^(^ ^38^ ^,^ ^62^ ^–^ ^64^ ^)^. Accordingly, considering the high HDL content of egg yolks, it is reasonable to hypothesise that consumption of carotenoid-enriched eggs could offer a unique delivery vehicle for gastrointestinal absorption and subsequent bioavailability for uptake and capture by target tissues (including retina and brain). In previous work, we supplemented the feed of Goldline hens with oil-based L, Z and MZ formulations, which resulted in the production of macular carotenoid-enriched eggs containing L and MZ in a 1:1 ratio in their yolks^(^ ^65^ ^)^.

In the current study, known as the Egg Xanthophyll Intervention clinical Trial (EXIT), we report on the outcomes of feeding these eggs to human subjects in terms of serum concentrations of the macular carotenoids (and cholesterol), MP and visual function.

## Methods

### Study design and subjects

EXIT is a single-blind placebo-controlled 8-week clinical trial that studied the impact of macular carotenoid-enriched eggs on serum carotenoid concentrations, visual performance, MP and serum cholesterol levels in human subjects. All subjects signed an informed consent document, confirming their willingness to participate in the trial. Ethical approval for the trial was granted by the Ethical Committee of the Waterford Institute of Technology (WIT), Waterford, Ireland, and the trial also conformed to the tenets of the Declaration of Helsinki. The EXIT trial was registered on the website www.controlled-trials.com (registration number ISRCTN25867083) on the 9th of August 2013 before participant enrollment. The study was then initiated in September 2013 (first subject study visit) and completed in November 2013 (last subject study visit). For a graphical summary of the paper, see the CONSORT diagram (online Supplementary material).

The main outcome measures of the trial were serum carotenoid concentrations, MP, visual function and serum cholesterol levels.

Totally, fifty subjects between the ages of 18 and 65 years were recruited into the trial from the staff of WIT, at two different sites: site 1, the Tourism and Leisure building on the main WIT campus, and site 2, the Arc Labs building at the WIT west campus. Inclusion criteria for EXIT included the following: no known allergy to eggs, no history of CVD, no ocular pathology and cholesterol levels of ≤6·5 mmol/l. In addition, subjects with current or recent history of supplementation with the macular carotenoids and/or cholesterol-lowering statins were excluded from the trial. The fifty subjects were divided equally into two groups of twenty-five.

For two-tailed tests at the 5 % level of significance, a sample of this size has power of 0·97 to detect an effect size (the size of the mean difference between the two groups) of 0·8 sd (of the variable of interest) within groups (paired *t* test) and a power of 0·79 to detect an effect size of 0·8 sd between groups (independent samples *t* test); the sample does not have sufficient power to detect smaller effect sizes of, for example, 0·5 sd.

Group 1 was supplemented daily with a standard control (placebo) egg at site 1, on the main WIT campus, whereas group 2 was given a macular carotenoid-enriched egg (active intervention), containing L:MZ in a 1:1 ratio at site 2, on the WIT west campus. There was a 3-km distance between both study sites. Each group was based on a different campus site to avoid possible ‘contamination’ of egg samples between the two groups, and also to preserve the single-blind (masked) nature of the trial, as the macular carotenoid-enriched eggs had a more pronounced yellow colour than the control eggs, and these colour differences may have been discerned by participants if mixing of the two groups had been allowed. Supplementation periods were also staggered between the two groups to accommodate the completion of final visits within 1 week of ending the supplementation period, as testing all subjects in 1 week would not be logistically feasible at our research centre. As a result, participants were not randomly assigned to the two intervention groups. Vision testing was carried out on all subjects at baseline and final visits, whereas serum carotenoid and total cholesterol levels were examined at baseline, week 4 and final visit. Clinical assessments were conducted by J. D., a researcher who was suitably trained on all aspects of the EXIT protocols.

### Study supplement: carotenoid-enriched hen eggs

Production of the carotenoid-enriched eggs has been described previously^(^ ^65^ ^)^. In brief, 120 Goldline hens, of approximately 20 weeks of age, were divided into two groups of sixty hens. For the duration of the trial, the hens were housed in a purpose-built barn on a farm in County Kilkenny in Southern Ireland. This barn was tested and quality assured by the Food Safety Authority of Ireland, and complied with all health standards prescribed by the Irish Food Board (BordBía), including testing for the presence of salmonella. The first group of hens was fed a standard complete grain feed, without any additional carotenoids, whereas the second group was given the same standard grain feed as hens in group 1, but incorporating MZ and L in a 1:1 ratio (70 mg/kg of each), for the generation of a 140 mg/kg carotenoid-enriched feed. The feed was stored sealed (at room temperature), to prevent contamination, in 60-kg containers, and fresh feed was provided daily in the barns hen feeders.

Supplementation of the hens with the feed began 3 weeks before commencement of subject supplementation with laid eggs. This was done to allow the eggs to reach their yolk carotenoid saturation point. Any eggs collected between weeks 1 and 3 of hen supplementation were hence disposed of as they were not considered suitable as subject supplement eggs.

Eggs intended for subject supplementation were collected daily and labelled with group numbers to avoid cross-contamination between control eggs and carotenoid-enriched eggs. These eggs were then transported to site 1 and refrigerated at 5°C until they were cooked for consumption, which took place within 1 week of the egg collection. As a quality check, four eggs were sampled weekly from both trial groups and tested for yolk carotenoid concentrations, as described previously^(^ ^65^ ^)^.

With regard to the cooking of the eggs used in the EXIT trial, all eggs were prepared by one chef, and scrambling was chosen as the cooking method following the assessment of various cooking options such as boiling, frying and scrambling, as scrambling of the eggs yielded similar concentrations of carotenoids as the other cooking methods but was considered more convenient logistically. To ensure reproducibility, a two-egg scrambled portion was measured using a standardised cup for each individual subject portion. This cooking method was selected for the comparable dosage that could be achieved between subjects when the eggs were prepared in batch form, and also for logistical reasons, namely for the ease of transportation of the group 2 eggs to site 2. This was achieved in a temperature-controlled ‘hot box’, which kept the temperature of the eggs constant (<10°C drop) during transit. To account for any tedium that may be experienced because of the necessity for daily consumption of scrambled eggs for an 8-week study period, different side options were served along with the eggs. Coffee, tea and orange juice were served with breakfast each day, with toast as a side option on Mondays, Wednesdays and Fridays, croissants on Tuesdays and English muffins on Thursdays. A study investigator attended both sites for the duration of the trial to monitor compliance, and in the event that any subjects were absent two eggs were given to them to take home and prepare themselves either that day or on a weekend day to account for their day of absence on site. This ensured that 100 % compliance was achieved with all subjects. Eggs were not consumed on weekend days (Saturday and Sunday) as a normal part of the trial.

### Macular pigment measurement

MP was measured at baseline and at final visit (8 weeks) by both customised heterochromatic flicker photometry using the Macular Densitometer (Macular Metrics Corp.)^(^ ^66^ ^,^ ^67^ ^)^ and by dual-wavelength autofluorescence using the Spectralis HRA+OCT Multicolour (Heidelberg Engineering GmbH). Detailed descriptions of the techniques for MP measurement by both the Densitometer^(^ ^68^ ^,^ ^69^ ^)^ and the Spectralis^(^ ^70^ ^,^ ^71^ ^)^ have been previously reported^(^ ^28^ ^)^. For the purposes of the current clinical trial, MP at 0·25, 0·5 and 1·0° of retinal eccentricity (Densitometer) and MP at 0·23, 0·51 and 1·02°, in addition to total MP volume (Spectralis), all with a reference point at 7°, are reported.

### Visual function assessment

Visual function of the EXIT subjects was assessed by contrast sensitivity (CS) and best-corrected visual acuity (BCVA). BCVA was measured with a computerised Early Treatment Diabetic Retinopathy Study (ETDRS) logarithm of the minimum angle of resolution (LogMAR) test chart (Test Chart 2000 Xpert; Thomson Software Solutions) at a distance of 4 m. Letter CS was assessed using the computerised LogMAR ETDRS test chart (Test Chart 2000 PRO; Thomson Software Solutions) at five different spatial frequencies (1·2, 2·4, 6·0, 9·6, 15·15 cycles per degree (cpd)). In addition, CS was assessed using the Functional Vision Analyzer (Stereo Optical Co. Inc.)^(^ ^72^ ^)^, which uses the functional acuity contrast test (FACT) at five different spatial frequencies (1·5, 3, 6, 12, 18 cpd) and under the following light conditions: mesopic, photopic, mesopic with glare and photopic with glare. Detailed descriptions of these visual function techniques have been previously described^(^ ^73^ ^)^.

### Serum carotenoid analysis

Serum L and total zeaxanthin (TZ) (including Z, MZ and *cis*-zeaxanthin (CisZ)) concentrations were measured at baseline, trial midpoint (4 weeks) and at the final subject visit (8 weeks). Non-fasting blood samples were collected from study subjects at each visit, as previously described^(^ ^28^ ^)^, and stored at −80°C until the time of analysis. Serum carotenoid measurements were determined using two separate HPLC assays: assay 1, a reversed-phase HPLC assay, which quantified both L and TZ concentrations, as previously described^(^ ^74^ ^)^, and assay 2, a normal-phase assay, which exploited chiral chromatography to separate the Z and MZ enantiomers that were collected as a TZ peak in assay 1. A detailed description of HPLC assay 2 has also been described previously^(^ ^1^ ^)^.

### Egg yolk carotenoid analysis

Four eggs were removed each week from group 1 and group 2 egg batches and analysed for their carotenoid content. This was performed to investigate whether carotenoid levels remained constant in the supplemental eggs over the course of the trial. Preparation of the egg yolks, extraction of the carotenoids and their analysis by HPLC were performed as previously described^(^ ^65^ ^)^.

### Serum cholesterol analysis

#### Total cholesterol

Total cholesterol was measured using the handheld Roche Accutrend Plus instrument (Accutrend Cholesterol Cobas^®^ system, list number 11418262, 2014; Roche Diagnostics GmbH) and associated Roche cholesterol test strips (DocCheck; Amtsgericht Stuttgart), by the classical ‘finger prick’ method at baseline, trial midpoint (beginning of week 5) and at the final subject visit (8 weeks). Cholesterol levels were monitored in the EXIT trial to ensure that no subjects’ cholesterol level became elevated >6·5 mmol/l.

#### Serum HDL-cholesterol

HDL levels of non-fasting serum samples collected at the EXIT subjects study visits were measured by Claymon Laboratories Ltd (Biomnis Ireland). HDL-cholesterol was measured using the Abbott Architect Ultra N-geneous^®^ HDL assay (HDL-cholesterol: Abbott Architect Ultra HDL Instructions for Use, list number 3K33-21, August 2015; Abbott Laboratories).

#### Serum LDL-cholesterol

LDL levels of non-fasting serum samples collected at the EXIT subjects’ study visits were measured by Claymon Laboratories Ltd (Biomnis Ireland). LDL-cholesterol was measured using the MULTIGENT Direct LDL assay (LDL-cholesterol: Abbott Architect Direct LDL Instructions for Use, list number 1E31-20, August 2015; Abbott Laboratories).

#### Serum TAG

Serum TAG levels of non-fasting serum samples collected at the EXIT subjects’ study visits were measured by Claymon Laboratories Ltd (Biomnis Ireland). Serum TAG were measured using the Abbott Architect Triglyceride assay (Triglycerides: Abbott Architect Triglycerides reagent kit instructions for use, list number 7D74, December 2012; Abbott Laboratories).

### Statistical analysis

The statistical package IBM SPSS version 21 was used for all statistical analyses. Between-group differences (between control and carotenoid-enriched egg groups) at baseline were investigated using independent-sample *t* or *χ* ^2^ tests as appropriate, and any variables (such as age or sex) found to be significantly different between the two groups were controlled for in subsequent analyses. Changes over time in the primary and secondary outcome measures were analysed using both paired *t* tests (for within-group changes over time) and general linear models, the latter controlling for variables found to be significantly different between groups at baseline. The 5 % significance level was used throughout all analyses, without any adjustment for multiple tests.

## Results

### Subject dropouts and adverse events

Of the fifty subjects originally enrolled in the study, two were removed from the study at midpoint (4 weeks), as their measured cholesterol concentrations exceeded the predetermined upper threshold limit of 6·5 mmol/l. Two further subjects also withdrew from the trial for personal reasons. Therefore, forty-six subjects (twenty-three in each study group) successfully completed the trial. There were no adverse events reported by subjects in either study group over the course of the trial.

### Baseline differences between the two study groups

Table 1 presents the baseline demographic, health and lifestyle, MP, cholesterol and serum carotenoid data of the control egg group and carotenoid-enriched egg group subjects in the EXIT clinical trial. As presented in Table 1, the variables for which we found statistically significant differences between the study groups were age, sex and TAG levels, and hence we controlled for these in subsequent analyses, where appropriate.

**Table 1:** Baseline demographic, health and lifestyle, cholesterol, macular pigment (MP), visual function and serum carotenoid data of the control group and enriched-group subjects (Mean values and standard deviations for numerical data and percentages for categorical data)

	Control group (*n* 25)		Enriched-group (*n* 25)		
Variables	Mean	sd	Mean	sd	Sig.
Age (years)	41	10	35	8	0·015*
BMI (kg/m^2^)	25	3·6	25·7	3	0·478
Diet score (estimated L and Z intake)	20·5	14·6	21·5	9·6	0·776
Exercise (minimum (h)/week)	329·4	322·6	219·8	136·1	0·124
Smoking (%)					0·583
Never smoked	64		68		
Past smoker	16		20		
Current smoker	20		12		
Education (highest level %)					0·412
Primary	4		0		
Secondary	4		4		
Higher (third level)	92		96		
Sex (%)					0·001*
Male	40		84		
Female	60		16		
Baseline cholesterol (mmol/l)					
HDL-cholesterol	1·3	0·2	1·1	0·3	0·119
LDL-cholesterol	2·6	0·6	2·6	0·5	0·655
Total cholesterol	4·8	0·5	4·8	0·5	0·985
TAG	1·1	0·4	1·6	0·9	0·018*
Baseline MP					
Densitometer					
MP 0·25°	0·527	0·17	0·549	0·19	0·674
MP 0·5°	0·413	0·16	0·440	0·19	0·596
MP 1°	0·283	0·17	0·276	0·14	0·895
Spectralis					
MP 0·23°	0·475	0·13	0·521	0·18	0·319
MP 0·51°	0·378	0·11	0·414	0·16	0·369
MP 1·02°	0·271	0·08	0·272	0·13	0·981
MP volume	5042	2054	5070	2145	0·962
BCVA	105	4·5	106	5·6	0·579
CS					
Letter CS 1·2 cpd	2·929	4·10	1·924	0·26	0·247
Letter CS 2·4 cpd	1·984	0·14	1·918	0·24	0·281
Letter CS 6 cpd	1·707	0·17	1·651	0·29	0·427
Letter CS 9·6 cpd	1·490	0·21	1·435	0·31	0·486
Letter CS 15·15 cpd	1·114	0·24	1·121	0·31	0·932
M FACT CS 1·5 cpd	1·756	0·17	1·773	0·15	0·700
M FACT CS 3 cpd	1·881	0·21	1·858	0·15	0·646
M FACT CS 6 cpd	1·660	0·25	1·618	0·30	0·592
M FACT CS 12 cpd	1·141	0·35	1·214	0·36	0·468
M FACT CS 18 cpd	0·540	0·32	0·513	0·28	0·751
P FACT CS 1·5 cpd	1·713	0·19	1·725	0·15	0·797
P FACT CS 3 cpd	1·941	0·18	1·965	0·13	0·597
P FACT CS 6 cpd	1·909	0·24	1·914	0·24	0·935
P FACT CS 12 cpd	1·619	0·34	1·590	0·27	0·746
P FACT CS 18 cpd	0·906	0·37	0·984	0·32	0·434
MG FACT CS 1·5 cpd	1·529	0·20	1·423	0·27	0·128
MG FACT CS 3 cpd	1·665	0·21	1·559	0·31	0·165
MG FACT CS 6 cpd	1·307	0·32	1·327	0·29	0·818
MG FACT CS 12 cpd	0·871	0·31	0·912	0·32	0·650
MG FACT CS 18 cpd	0·332	0·11	0·325	0·08	0·801
PG FACT CS 1·5 cpd	1·624	0·14	1·642	0·18	0·699
PG FACT CS 3 cpd	1·923	0·13	1·923	0·11	0·994
PG FACT CS 6 cpd	1·829	0·23	1·860	0·24	0·647
PG FACT CS 12 cpd	1·515	0·38	1·566	0·23	0·569
PG FACT CS 18 cpd	0·906	0·36	1·033	0·25	0·150
Baseline serum carotenoids (µmol/l)					
Lutein	0·226	0·13	0·188	0·08	0·234
Zeaxanthin	0·079	0·04	0·074	0·02	0·531
*cis*-Zeaxanthin	0·024	0·01	0·026	0·01	0·588
*meso*-Zeaxanthin	–		–		–

Variables, variables analysed in the study; control group, subjects consuming normal eggs; enriched group, subjects consuming lutein and *meso*-zeaxanthin enriched eggs; Sig., the statistical difference (*P* value) between between control and enriched-group subjects assessed using either independent samples *t* tests or χ depending on the variable of interest; BMI, measure of body fat based on height and weight (i.e. the body mass divided by the square of the body height); diet score, estimated dietary intake of lutein and zeaxanthin; exercise, total exercise measured as minutes per week engaged in physical or sporting activity; smoking (%), current smoker (smoked ≥100 cigarettes in lifetime and at least one in the last year), past smoker (smoked ≥100 cigarettes in lifetime and none in the past year) or non-smoker (smoked <100 cigarettes in lifetime); education (highest level %), highest level to which the subject was educated; total cholesterol, measure of HDL, LDL and TAG levels; TAG, fat molecules found in the blood; esters composed of glycerol and three fatty acids; MP 0·25°, spatial profile of MP measured at 0·25° of retinal eccentricity with a reference point at 7° (measured using the macular densitometer); MP 0·5°, spatial profile of MP measured at 0·5° of retinal eccentricity with a reference point at 7° (measured using the Macular Densitometer^®^); MP 1°, spatial profile of MP measured at 1° of retinal eccentricity with a reference point at 7° (measured using the macular densitometer); MP 0·23°, macular pigment optical density at 0·23° of retinal eccentricity (measured using the Heidelberg Spectralis^®^ HRA+OCT MultiColour); MP 0·51°, macular pigment optical density at 0·51° of retinal eccentricity (measured using the Heidelberg Spectralis^®^ HRA+OCT MultiColour); MP 1·02°, macular pigment optical density at 1·02° of retinal eccentricity (measured using the Heidelberg Spectralis^®^); MP volume, a volume of MP calculated as MP average times the AUC out to 7° eccentricity (measured using the Heidelberg Spectralis^®^); BCVA, best-corrected visual acuity reported as visual acuity rating; CS, contrast sensitivity reported in LogCS; cpd, cycles per degree; M FACT, functional acuity contrast test under mesopic light conditions; P FACT, functional acuity contrast test under photopic light conditions; MG FACT, functional acuity contrast test under mesopic with glare light conditions; PG FACT, functional acuity contrast test under photopic with glare light conditions; baseline serum carotenoids, serum concentrations of lutein, zeaxanthin, *cis*-zeaxanthin and *meso*-zeaxanthin (µmol/l). * Statistically significant differences between control and enriched-group subjects.

### Within-group changes over time (paired-sample *t* tests)

The first research question addressed was as follows: which study variables changed significantly over the 8-week study period? Table 2 displays, separately for the intervention and control groups, the results of paired *t* test analyses for all MP, serum, cholesterol and vision variables. Statistically significant differences in the table are indicated using asterisk.

**Table 2:** Within-group changes over time (paired-sample *t* tests) data for (a) macular pigment (MP), serum carotenoids (lutein (L), total zeaxanthin (TZ), *cis*-zeaxanthin (CisZ), zeaxanthin (Z) and *meso*-zeaxanthin (MZ), cholesterol and TAG (b) contrast sensitivity (CS) and visual acuity (VA) in the control and enriched egg groups† (Mean values and standard deviations)

	Baseline visit		Final visit			
Variables and study group	Mean	sd	Mean	sd	Diff.	Sig.
*(a) MP, serum carotenoids (L, TZ, CisZ, Z and MZ), cholesterol and TAG*						
MP 0·25						
Control	0·527	0·18	0·542	0·21	0·015	0·507
Enriched	0·554	0·20	0·531	0·23	−0·022	0·448
MP 0·5						
Control	0·410	0·17	0·437	0·20	0·027	0·186
Enriched	0·446	0·20	0·411	0·21	−0·035	0·244
MP 1·0						
Control	0·304	0·17	0·304	0·15	0·000	0·980
Enriched	0·283	0·15	0·228	0·19	−0·054	0·158
MP 0·23						
Control	0·470	0·13	0·485	0·15	0·015	0·250
Enriched	0·528	0·19	0·759	1·16	0·230	0·333
MP 0·51						
Control	0·378	0·12	0·390	0·13	0·012	0·279
Enriched	0·421	0·17	0·421	0·17	0·000	0·921
MP 1·02						
Control	0·277	0·09	0·283	0·09	0·006	0·163
Enriched	0·280	0·14	0·279	0·14	0·001	0·589
MP volume						
Control	5215·91	2024·46	5276·09	2001·63	60·182	0·600
Enriched	5216·64	2213·73	5245·55	2253·64	28·91	0·764
Serum L (µmol/l)						
Control	0·228	0·13	0·298	0·18	0·070	0·007*
Enriched	0·195	0·08	0·441	0·15	0·246	<0·001*
Serum TZ (µmol/l)						
Control	0·080	0·04	0·111	0·05	0·031	0·009*
Enriched	0·074	0·02	0·208	0·08	0·134	<0·001*
Serum CisZ (µmol/l)						
Control	0·025	0·01	0·048	0·02	0·023	<0·001*
Enriched	0·027	0·01	0·094	0·03	0·067	<0·001*
Serum Z (µmol/l)						
Control	0·080	0·04	0·111	0·05	0·031	0·009*
Enriched	0·074	0·02	0·124	0·05	0·050	<0·001*
Serum MZ (µmol/l)						
Control	–	–	–		–	–
Enriched	–	–	0·084	0·04	0·084	<0·001*
Total cholesterol (mmol/l)						
Control	4·77	0·52	5·19	0·63	0·420	0·003*
Enriched	4·76	0·45	4·981	0·55	0·220	0·025*
HDL-cholesterol (mmol/l)						
Control	1·26	0·25	1·31	0·27	0·051	0·112
Enriched	1·14	0·29	1·09	0·26	−0·054	0·065
LDL-cholesterol (mmol/l)						
Control	2·58	0·54	2·62	0·63	0·038	0·664
Enriched	2·52	0·47	2·59	0·55	0·070	0·444
TAG (mmol/l)						
Control	1·11	0·46	1·19	0·69	0·083	0·366
Enriched	1·61	0·91	1·54	0·72	−0·070	0·631
*(b) CS and VA*						
Letter CS 1·2 cpd						
Control	2·045	0·11	1·965	0·12	−0·080	0·017*
Enriched	1·970	0·13	2·009	0·11	0·039	0·334
Letter CS 2·4 cpd						
Control	1·968	0·14	1·968	0·13	0·000	1·000
Enriched	1·966	0·12	1·965	0·12	0·001	0·971
Letter CS 6 cpd						
Control	1·704	0·17	1·684	0·19	−0·020	0·585
Enriched	1·697	0·22	1·712	0·17	0·015	0·834
Letter CS 9·6 cpd						
Control	1·478	0·22	1·466	0·21	−0·012	0·801
Enriched	1·484	0·25	1·541	0·21	0·057	0·508
Letter CS 15·15 cpd						
Control	1·102	0·24	1·095	0·30	−0·007	0·830
Enriched	1·182	0·25	1·140	0·23	−0·042	0·703
M FACT CS 1·5 cpd						
Control	1·754	0·17	1·740	0·17	−0·013	0·745
Enriched	1·789	0·15	1·743	0·17	−0·046	0·189
M FACT CS 3 cpd						
Control	1·879	0·22	1·939	0·15	0·059	0·186
Enriched	1·878	0·13	1·832	0·28	−0·046	0·356
M FACT CS 6 cpd						
Control	1·654	0·26	1·691	0·24	0·037	0·489
Enriched	1·647	0·30	1·658	0·29	0·011	0·851
M FACT CS 12 cpd						
Control	1·138	0·36	1·144	0·36	0·006	0·902
Enriched	1·238	0·36	1·163	0·32	−0·075	0·025*
M FACT CS 18 cpd						
Control	0·548	0·33	0·520	0·29	−0·027	0·597
Enriched	0·519	0·29	0·548	0·31	0·029	0·697
P FACT CS 1·5 cpd						
Control	1·701	0·19	1·702	0·18	0·008	0·982
Enriched	1·735	0·15	1·722	0·15	−0·013	0·556
P FACT CS 3 cpd						
Control	1·938	0·18	1·990	0·13	−0·052	0·119
Enriched	1·980	0·09	1·994	0·11	0·014	0·532
P FACT CS 6 cpd						
Control	1·898	0·25	1·890	0·42	−0·008	0·928
Enriched	1·935	0·22	1·916	0·20	−0·019	0·575
P FACT CS 12 cpd						
Control	1·611	0·35	1·669	0·28	0·058	0·205
Enriched	1·597	0·28	1·717	0·24	0·120	0·067
P FACT CS 18 cpd						
Control	0·912	0·38	0·997	0·37	0·085	0·222
Enriched	0·995	0·34	0·978	0·36	−0·017	0·761
MG FACT CS 1·5 cpd						
Control	1·539	0·20	1·501	0·21	−0·038	0·372
Enriched	1·439	0·28	1·539	0·23	0·100	0·047*
MG FACT CS 3 cpd						
Control	1·664	0·22	1·647	0·25	−0·017	0·646
Enriched	1·594	0·27	1·637	0·24	0·043	0·315
MG FACT CS 6 cpd						
Control	1·302	0·33	1·302	0·38	0·000	0·993
Enriched	1·343	0·28	1·318	0·32	−0·025	0·639
MG FACT CS 12 cpd						
Control	0·895	0·31	0·923	0·35	0·029	0·612
Enriched	0·934	0·32	0·907	0·28	−0·027	0·604
MG FACT CS 18 cpd						
Control	0·335	0·12	0·356	0·15	0·021	0·328
Enriched	0·328	0·09	0·342	0·14	0·014	0·715
PG FACT CS 1·5 cpd						
Control	1·610	0·14	1·675	0·17	0·065	0·066
Enriched	1·660	0·17	1·694	0·19	0·034	0·143
PG FACT CS 3 cpd						
Control	1·912	0·12	1·952	0·17	0·040	0·201
Enriched	1·932	0·10	2·545	2·76	0·612	0·311
PG FACT CS 6 cpd						
Control	1·831	0·24	1·898	0·24	0·068	0·144
Enriched	1·888	0·20	1·895	0·24	0·007	0·826
PG FACT CS 12 cpd						
Control	1·530	0·37	1·645	0·33	0·115	0·016*
Enriched	1·585	0·24	1·626	0·25	0·041	0·428
PG FACT CS 18 cpd						
Control	0·925	0·35	0·928	0·39	0·003	0·949
Enriched	1·052	0·27	1·047	0·40	−0·005	0·940
BCVA						
Control	105·217	4·66	105·35	4·78	0·130	0·761
Enriched	106·227	5·15	107·73	4·45	1·50	0·074

Variable, variables analysed in the study; control, normal egg study group; enriched, carotenoid-enriched egg study group; baseline visit, first subject visit at study initiation; final visit, final subject visit at trial end point (8 weeks); Diff., difference in variable values between the final study visit and the baseline study visit; Sig., the statistical difference (*P* value) between the baseline study visit and final study assessed using paired-sample *t* tests; CS, contrast sensitivity reported in LogCS; cpd, cycles per degree; M FACT, functional acuity contrast test under mesopic light conditions; P FACT, functional acuity contrast test under photopic light conditions; MG FACT, functional acuity contrast test under mesopic with glare light conditions; PG FACT, functional acuity contrast test under photopic with glare light conditions; BCVA, best-corrected VA reported as visual acuity rating. * Statistically significant differences between baseline and final study visits.

† Data displayed are the results of paired *t* test analyses.

### Between-group changes over time (repeated measures)

**Fig. 1:**
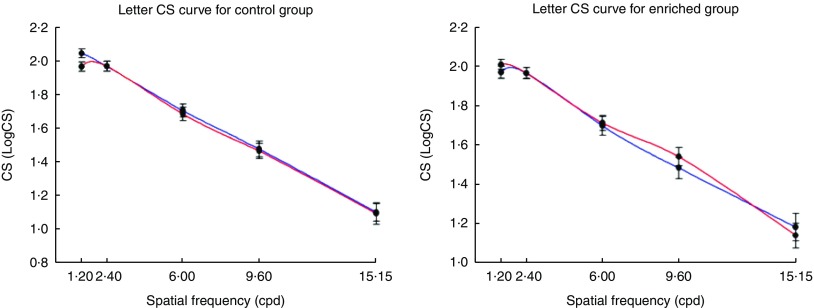
Change in letter contrast sensitivity (CS) values between baseline and final visit (8 weeks) and at five different spatial frequencies: 1·20, 2·40, 6·00, 9·60 and 15·15 cycles per degree (cpd) using the Test Chart 2000 PRO^TM^ (Thomson Software Solutions) in the Egg Xanthophyll Intervention Trial; control egg group subjects; one standard egg per day. Enriched egg group subjects; one lutein:*meso*-zeaxanthin enriched egg per day. An improvement in CS at final visit in the enriched egg group relative to the control group is seen at 15·15 cpd reflected in the higher LogCS value. Values are means, with their standard errors. 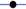, Baseline visit; 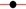, 8-week visit.

**Fig. 2:**
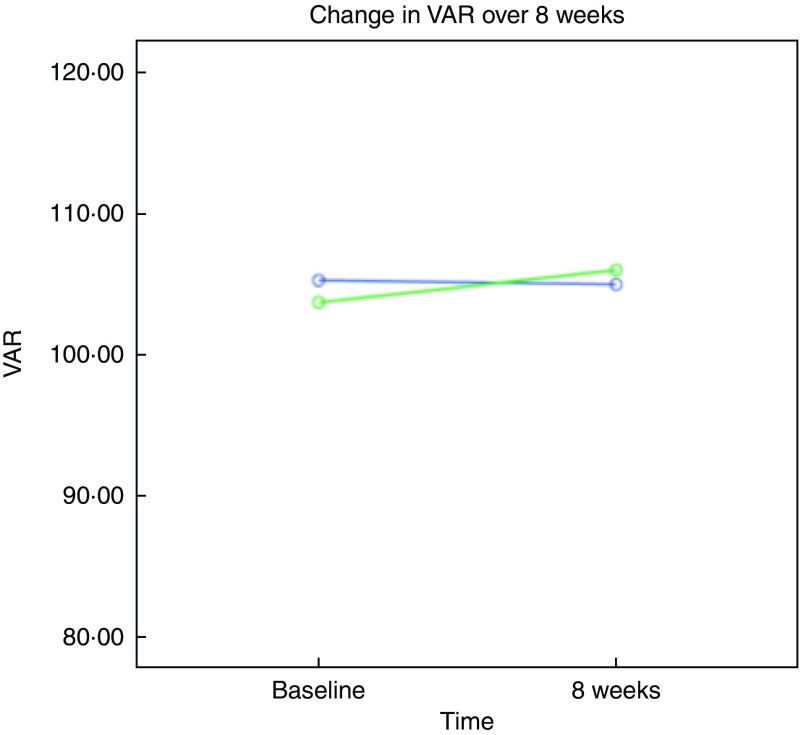
Change in best corrected visual acuity (BCVA) rating values between baseline and final visit (8 weeks) measured with Test Chart 2000 Xpert (Thomson Software Solutions) in the Egg Xanthophyll Intervention Trial; control egg group subjects (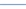); one standard egg per day. Enriched egg group subjects (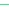); one lutein:*meso*-zeaxanthin enriched egg per day. An improvement in BCVA at final visit in the enriched egg group relative to the control group is reflected in the higher visual acuity rating (VAR).

**Fig. 3:**
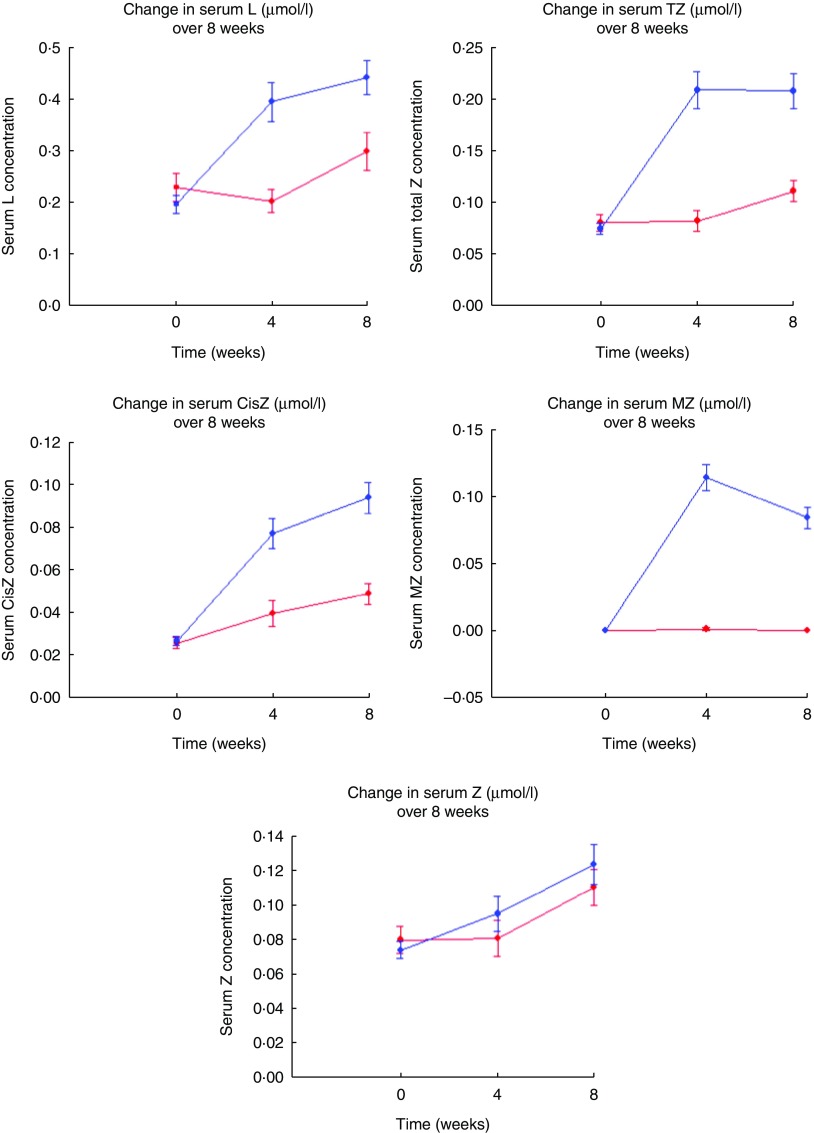
Change in serum concentrations (µmol/l) of lutein (L), total zeaxanthin (TZ), *cis*-zeaxanthin (CisZ), *meso*-zeaxanthin (MZ) and zeaxanthin (Z) between baseline, midpoint (4 weeks) and final visit (8 weeks) using both reversed phase HPLC for L, TZ and CisZ analysis, and normal phase HPLC for Z:MZ ratio analysis on an Agilent 1260 Series system (Agilent Technologies Limited) in the Egg Xanthophyll Intervention Trial; control egg group subjects (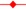); one standard egg per day. Enriched egg group subjects (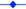); one L:MZ enriched egg per day. Increases in serum carotenoid levels can be seen at midpoint and final visits in the enriched egg group relative to the control group for L, TZ, CisZ and MZ, which are reflected in the higher concentration values seen. Increases in serum Z levels can be seen at midpoint in the enriched egg group relative to the control group, which are reflected in the higher concentration values seen. However, concentrations of Z were not significantly different between groups at final visit, reflected in the similarity of serum Z concentrations in both groups. Values are means, with their standard errors.

**Fig. 4:**
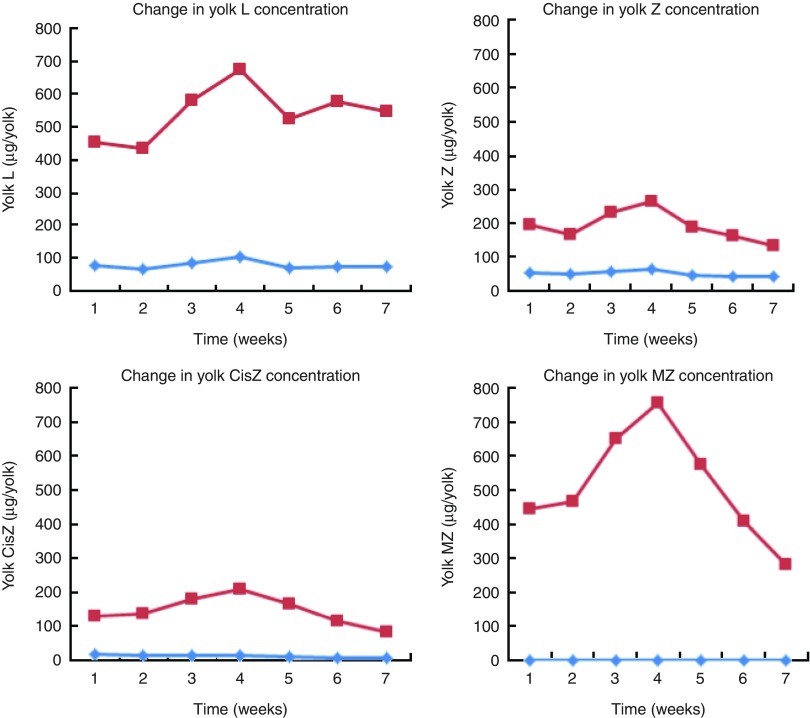
Weekly analysis of egg yolk concentrations (µg/yolk) of lutein (L), total zeaxanthin (TZ), *cis*-zeaxanthin (CisZ) and *meso*-zeaxanthin (MZ) over a 7-week period using both reversed phase HPLC for L, TZ and CisZ analysis, and normal phase HPLC for MZ analysis on an Agilent 1260 Series system (Agilent Technologies Limited) in the Egg Xanthophyll Intervention Trial. Week-to-week variation can be seen by analysis of the trend in concentration values of both the control (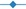) and enriched (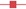) eggs.

The second research question addressed was as follows: which study variables changed significantly more in the enriched egg group compared with the control group? Repeated-measures ANOVA was used in this part of the study, to compare change in these variables between intervention and control groups. These analyses controlled for baseline age, TAG (except when the outcome variable was cholesterol related) and sex, all of which were significantly different between intervention and control groups at baseline. Results from analyses, within-group and between-group, are presented (below) for each outcome variable. Fig. 1–4 graphically illustrate the observed statistically significant between-group changes in outcome variables over the 8-week study period.

### Macular pigment measurement

MP was measured at baseline and at the final study visit (8 weeks) for both subject groups. As presented in Table 2, we found no significant MP response to egg supplementation in either the control group or the enriched egg group over the 8-week study period, nor were there significant between-group differences in MP at any measured eccentricities, whether measured on the Densitometer (MP 0·25; *P*=0·840, MP 0·5; *P*=0·593, MP 1·0; *P*=0·579) or Spectralis (MP 0·23; *P*=0·706, MP 0·51; *P*=0·663, MP 1·02; *P*=0·345, MP volume; *P*=0·979).

### Contrast sensitivity and best-corrected visual acuity

CS was measured at baseline and at the final study visit (8 weeks) for both subject groups. In relation to the within-group changes over the 8-week study period, presented in Table 2, we noted a significant decrease in the control group for letter CS at 1·2 cpd (*P*=0·017) and a significant increase in FACT CS at 12 cpd under photopic conditions with glare (*P*=0·016). In the enriched egg group, there was a significant decrease in FACT CS at 12 cpd under mesopic conditions (*P*=0·025), and a significant increase in FACT CS at 1·5 cpd under mesopic conditions with glare (*P*=0·047).

Controlling for age, sex and TAG using the general linear model repeated measures test, we noted only one between-group difference over the course of the study for measures of visual function, as presented in Fig. 1. This was for the letter CS at 15·15 cpd (*P*=0·046), which exhibited an improvement in the enriched egg group.

BCVA was measured at baseline and at the final study visit (8 weeks) for both subject groups. As presented in Table 2, there was no significant change in BCVA in either the control (*P*=0·761) or enriched (*P*=0·074) egg groups over the course of the study. However, we did note a statistically significant between-group difference (*P*=0·035) after controlling for age, sex and TAG using the general linear model repeated-measures test, because of a small improvement in BCVA in the enriched egg group and a small decrease in the control group (see Fig. 2).

### Serum carotenoid analysis

Serum carotenoid concentrations were measured at baseline, trial midpoint (4 weeks) and at the final subject visit (8 weeks). As presented in Table 2, serum L, TZ, Z and CisZ concentrations increased significantly over time in both the control (*P*=0·007, 0·009, 0·009 and <0·001, respectively) and enriched (*P*<0·001 for all) egg groups, whereas serum MZ concentration increased significantly only in the enriched egg group (*P*<0·001). In terms of between-group differences, controlling for age, sex and TAG using the general linear model repeated measures test, the enriched egg group showed a significantly greater serum response to L, TZ, CisZ and MZ (*P<*0·001 for all) than the control group. Both study groups were found to respond comparably with respect to Z, with no significant between-group difference noted after 8 weeks (*P*=0·477) (see Fig. 3).

### Egg yolk carotenoid analysis

Four eggs were taken weekly from the batch of study eggs of both normal and enriched egg groups and tested for their carotenoid content for the duration of the EXIT study. These results are presented in Fig. 4. Week-to-week variation in yolk L concentrations was not statistically significant over the course of the trial in either the control (*P*=0·258) or enriched (*P*=0·126) egg groups (*P* values quoted are from ANOVA). However, there was significant week-to-week variation in yolk Z (*P*=0·032), CisZ (*P*=0·010) and MZ (*P*=0·005) concentrations in the enriched egg group.

### Serum cholesterol analysis

Total cholesterol, HDL-cholesterol and TAG levels were measured at baseline, midpoint (4 weeks) and final study visit (8 weeks) for both subject groups. As presented in Table 2, total cholesterol levels increased significantly within both the control (*P*=0·003) and enriched (*P*=0·025) egg groups over the course of the study. However, we saw no statistically significant between-group differences for total cholesterol (*P*=0·561). Similarly, as presented in Table 2, we report no significant increase (*P*>0·05 for all), either within treatment groups or between treatment groups, in terms of HDL-cholesterol, LDL-cholesterol or TAG levels over the course of the trial.

## Discussion

This study presents findings of the EXIT, a clinical trial designed to study the impact of the consumption of normal (control) eggs, and carotenoid (L and MZ)-enriched eggs on serum carotenoid concentrations, visual performance and MP densities in human subjects. As a secondary outcome measure, serum cholesterol levels were also monitored in both study groups over the course of the trial. The rationale and motivation for undertaking the current study relates to the suggested role that the yolk matrix, which is liquid in nature and of high lipoprotein content, may play in enhancing carotenoid absorption and, therefore, bioavailability of these compounds in humans^(^ ^48^ ^,^ ^54^ ^)^. Indeed, the potential of eggs to enhance carotenoid serum responses, when compared with other foods and supplements, has been suggested by the findings of previous studies^(^ ^38^ ^,^ ^55^ ^,^ ^56^ ^)^. It is worth noting that previous studies have investigated the consumption of both L^(^ ^48^ ^,^ ^54^ ^,^ ^55^ ^,^ ^59^ ^,^ ^63^ ^,^ ^75^ ^)^- and Z^(^ ^62^ ^,^ ^76^ ^)^-enriched eggs and their effect on MP, vision and serum concentrations of the carotenoids. In addition, there was also one study that investigated the serum response to MZ-enriched eggs^(^ ^38^ ^)^. However, the current investigation is the first to report on the serum, MP and visual response to eggs enriched with L and MZ.

The main finding from this study was that there was a statistically significant increase in serum carotenoid concentrations in the control and enriched egg groups over the course of the trial, whereas no significant MP changes were seen in either study group. In relation to visual performance, although we noted some significant trends for both CS and VA measurements (in terms of some significant, but not clinically meaningful, improvements in the enriched egg group), overall, consumption of both the control and enriched eggs over the study period appeared to show no significant effects on subject’s visual performance. A significant increase in total cholesterol was noted in both control and enriched egg groups over the course of the trial; however, no significant changes in serum HDL and LDL or TAG levels were evident.

In relation to serum carotenoid changes in this study, it was perhaps not surprising that we saw a response in both the control and enriched egg study groups, as hen eggs are known to be naturally bioavailable sources of both L and Z because of the colocalisation of these xanthophylls with egg yolk HDL^(^ ^59^ ^,^ ^60^ ^,^ ^75^ ^)^. The enriched eggs used in this study induced a response in terms of serum L concentrations that was significantly greater than that seen among subjects supplemented with normal control eggs (increases of 126 and 31 %, respectively). The serum Z response was also greater (but not significantly so) in the enriched egg group when compared with the control group (68 and 39 % increases, respectively). These results are comparable with most^(^ ^48^ ^,^ ^54^ ^,^ ^63^ ^,^ ^75^ ^–^ ^78^ ^)^, but not all^(^ ^62^ ^,^ ^79^ ^,^ ^80^ ^)^, previous reports (Table 3). The finding by Wenzel *et al*.^(^ ^62^ ^)^ that serum Z, but not serum L, increased following 12 weeks’ supplementation could perhaps be considered unusual, as most studies (including the current one) demonstrate a clear increase in serum L concentrations following egg intervention. It is perhaps worth noting, however, that a more significant increase in serum Z was indeed noted in many studies when compared with that of serum L^(^ ^48^ ^,^ ^54^ ^,^ ^63^ ^,^ ^75^ ^,^ ^76^ ^)^. A possible contributory factor may be the effect of cooking of the eggs, as cooking-mediated losses of L have been reported to be greater than those of Z^(^ ^81^ ^)^. Thurnham^(^ ^38^ ^)^ has reported that observed increases in plasma Z concentrations can be a function of lower baseline Z concentrations in comparison with baseline L concentrations, thereby favouring more marked rises in serum concentrations of Z.

**Table 3:** Studies presenting the serum carotenoid and macular pigment (MP) response to egg supplementation

Study	Year	*n*	Sex	Age (years)	Study duration	Intervention	L response	Z response	MP response
Handelman *et al*.^(^ ^48^ ^)^	1999	11	6m, 5f	46–78	4·5 weeks/diet	G1: maize oil	G1: no response	G1: no response	–
					(washout period of ≥2 weeks between diets)	G2: beef tallow	G2: no response	G2: no response	–
						G3: maize oil+1·3 egg yolks daily	G3: 50 % increase relative to G1	G3: 114 % increase relative to G1	–
						G4: beef tallow+1·3 egg yolks daily	G4: 28 % increase relative to G2	G4: 142 % increase relative to G2	–
Goodrow *et al*.^(^ ^54^ ^)^	2006	33	7m, 26f	60–96	8 weeks	One egg daily	26 % increase	38 % increase	–
Wenzel *et al*.^(^ ^62^ ^)^	2006	24	0m, 24f	24–59	12 weeks	6 (L and Z in combination) enriched eggs weekly			Sig. increase in both treatment groups, in a linear manner from 4 to 8 weeks, to 12 weeks
						G1: 331 µg combination	G1: no sig response	G1: 50 % increase	
						G2: 946 µg combination	G2: no sig response	G2: 82 % increase	
Vishwanathan *et al*.(^63^)	2009	52	21m, 31f	≥60	5 weeks/phase	G1: 2 egg yolks daily	G1: 16 % increase	G1: 24 % increase	Changes in MP were inversely related to baseline MP levels
					(4-week egg-free period between phases)	G2: 4 egg yolks daily	G2: 36 % increase	G2: 82 % increase	G1: 30 % increase at 0·5° eccentricity
									G2: ≤50 % increase at 0·25, 0·5 and 1° eccentricity
van der Made *et al*.^(^ ^64^ ^)^	2016	100	32m, 68f	62–63	1 year	G1: egg-yolk-free buttermilk drink daily G2: 1·5 egg yolk buttermilk drink enriched with L, Z and DHA daily	G1: no sig response G2: 94 % increase	G1: no sig response G2: similar increase to L	G1: no sig response G2: sig. increase in L group relative to control group, and also within L group in comparison with baseline
Blesso *et al*.^(^ ^75^ ^)^	2013	37	12m, 25f	30–70	12 weeks	G1: egg-yolk-free substitute daily	G1: no sig response	G1: no sig response	–
						G2: 3 whole eggs daily	G2: 21 % increase	G2: 48 % increase	–
Kelly *et al*.^(^ ^76^ ^)^	2014	100	43m, 57f	≥18	90 d	G1: unmodified diet	G1: no sig response	G1: no sig response	No sig response
						G2: 1 normal egg daily	G2: no sig response	G2: no sig response	No sig response
						G3: 1 L-enriched egg daily	G3: 76 % increase	G3: no sig response	No sig response
						G4: 1 Z-enriched egg daily	G4: no sig response	G4: 430 % increase	No sig response
						G5: 1 L-enriched egg yolk beverage daily	G5: 77 % increase	G5: no sig response	No sig response
Mutungi *et al*.^(^ ^77^ ^)^	2010	31	31m, 0f	40–70	12 weeks	G1: egg-yolk-free substitute daily	G1: –	G1: 40 % decrease	–
						G2: 3 liquid eggs daily	G2: 80 % increase	G2: 40 % increase	–
van der Made *et al*.^(^ ^78^ ^)^	2014	89	29m, 60f	≥50	1 year	G1: egg-yolk-free buttermilk beverage daily	G1: no sig response	G1: no sig response	–
						G2: 1·5 egg yolk L-enriched buttermilk beverage daily	G2: 83 % increase relative to G1	G2: 110 % increase relative to G1	–
Surai *et al*.^(^ ^79^ ^)^	2000	44	24m, 20f	26–59	8 weeks	G1: 1 normal egg daily	G1: no sig response	G1: no sig response	–
						G2: 1 L-enriched egg daily	G2: 1·88-fold increase relative to G1	G2: no sig response	–
Bunger *et al*.^(^ ^80^ ^)^	2014	94	21m, 73f	18–35	6 weeks	G1: L-free 1·5 egg yolk buttermilk beverage	G1: 11·8 % decrease	G1: 18 % decrease	–
						G2: fresh L-enriched 1·5 egg yolk buttermilk beverage	G2: 107 % increase	G2: 55·1 % increase	–
						G3: dried version of G2 beverage suspended in water	G3: 51·2 % increase	G3: 48·1 % increase	–
						G4: dried individual components of G2 beverage suspended in water	G4: 70·9 % increase	G4: 56·8 % increase	–
EXIT study: Kelly *et al*.*	2016	50	31m, 19f	18–65	8 weeks	G1: 2 scrambled eggs daily	G1: 31 % increase	G1: 39 % increase	No sig response
						G2: 2L and MZ-enriched scrambled eggs daily	G2: 126 % increase	G2: 68 % increase	No sig response

*n*, Number of subjects; m, male; f, female; L, lutein; Z, zeaxanthin; –, not measured; G, study group; sig, significant; EXIT, Egg Xanthophyll Intervention Trial; MZ, *meso*-zeaxanthin.

* MZ levels increased significantly (*P*<0·001) in this study from baseline to final visit in the enriched egg group (MZ was not measured in the other studies). All increases or decreases are calculated from the baseline levels unless otherwise stated.

As MZ is not present in non-enriched eggs, and no other studies investigating the consumption of MZ-enriched eggs have been published, it is difficult to discuss the between-intervention-group response to MZ in our study. However, the serum MZ response from the enriched eggs (0·118 µmol/l per mg) in the current study is comparable with that achieved in oil-based supplementation using commercially available formulations (0·004 and 0·005 µmol/l per mg, respectively)^(^ ^1^ ^,^ ^82^ ^)^, but with a considerably lower dosage (0·718 mg, as opposed to 10 and 10 mg, respectively), and is also greater than the response (0·026 µmol/l per mg) reported by Thurnham *et al.* ^(^ ^83^ ^)^, achieved using an 8-mg supplement. Interestingly, in our study, in the enriched egg group, observed rises in serum concentrations of CisZ (which is likely to be a combination of *cis*-Z and *cis*-MZ) were similar to the observed rises in serum MZ (Fig. 3), despite the considerably higher concentrations of MZ compared with CisZ in the raw control egg yolks (Fig. 4). There may be several reasons for this observation, including the following: (1) a portion of yolk MZ (and also possibly yolk Z) may be metabolised to its respective *cis* form during uptake or absorption, hence contributing to the overall CisZ response – indeed, previous reports have noted augmentation of serum CisZ in response to supplementation with Z^(^ ^84^ ^,^ ^85^ ^)^; (2) there may have been thermally induced isomerisation of *trans*-Z and MZ during the cooking of the eggs, as this has been previously reported in the case of some vegetables, including maize^(^ ^86^ ^)^.

With respect to the relationship between serum concentrations arising from egg consumption and MP in our study, we found no significant correlations in either study group over the 8-week intervention period. This finding is in agreement with one previous report^(^ ^76^ ^)^, but in contrast to that of others^(^ ^62^ ^–^ ^64^ ^)^ (Table 3). When discussing the MP findings in the current study, it is perhaps worthwhile to note the study of Broekmans *et al.* ^(^ ^87^ ^)^. Interestingly, in that study, in which cross-sectional data were analysed, the authors found that in a group of 376 subjects (which were almost equally split as regards sex), MP levels were 13 % higher in men than in women. In contrast, serum carotenoid concentrations and adipose tissue concentrations of L were significantly higher in women (however, it is known that there is a higher concentration of adipose tissue in the body composition of women than in men^(^ ^88^ ^)^, and therefore this may naturally have influenced greater L absorption by adipose tissue in women). The observation that men exhibited lower concentrations of L in serum and in adipose tissue, and yet higher MP, suggests that men would likely be more responsive to attempts to augment MP through dietary modification, and that adipose tissue appears to compete for L with MP’s constituent carotenoids, as suggested previously^(^ ^89^ ^)^. Hence, it may be important to consider adiposity when reporting the relationship between carotenoid intake and MP. In relation to the current study, the sex split was 84 % male and 16 % female in the enriched egg group, and 40 % male and 60 % female in the control egg group. The fact that we did not see a change in MP in the enriched egg group in the current study is perhaps unusual, given the predominance of males in that group. In this regard, it may also be important to highlight that concentrations of carotenoids in serum reflect more recent dietary intake, whereas adipose tissue concentrations more accurately reflect longer-term dietary intake of carotenoids^(^ ^89^ ^)^, and therefore in a shorter study (such as the current one) serum and adipose tissue effects on MP density may not have been elicited.

With regard to visual performance, in our study, the small number of statistically significant results for letter CS (i.e. one’s ability to discriminate the foreground from the background) and VA (i.e. sharpness of vision at 100 % contrast), which we noted in the enriched egg group, should be assessed with caution. First, none of the improvements were clinically significant and, second, the statistical significance that we report (e.g. for four CS measures in Table 2) may well be a consequence of multiple testing.

An improvement in CS without an increase in MP has been noted in a previous study by our group^(^ ^20^ ^)^, although this was evident at 6 months post intervention. Overall, the lack of improvement in visual performance of the EXIT subjects is consistent with the lack of MP augmentation also observed, as it has been shown that CS improvements are typically commensurate with observed augmentations in MP^(^ ^20^ ^)^. It is also important to note that, as seen in Table 1, the baseline values for BCVA and CS in both subject groups in our study were considered high, and therefore our finding (of limited clinically meaningful improvements in their visual performance) is not unexpected, particularly considering the relatively short duration of the trial. Indeed, we have previously shown that improvements in visual performance in healthy subjects without AMD are possible over a longer duration (12 months)^(^ ^21^ ^)^. In addition, in the current study, we considered whether low and high carotenoid responders may have behaved differently in terms of their visual parameters. However, this would have reduced group sizes significantly, and after a cursory examination we felt that such an analysis would be unjustified.

We showed that total cholesterol levels increased significantly over the 8-week trial period in both the control egg (9 % increase) and enriched egg (5 % increase) groups, but upper limits of the normative reference values (6·5 mmol/l) were not exceeded, and this finding is consistent with previous studies, where total cholesterol increases of 4 %^(^ ^90^ ^)^ and 5 %^(^ ^91^ ^)^ were reported. Moreover, we found no significant increases in LDL, HDL or TAG levels in either study group, consistent with other egg-based studies^(^ ^54^ ^,^ ^62^ ^,^ ^80^ ^)^.

The limitations of this study include the relatively short study period and small sample size, the lack of randomisation of the treatment groups and the high male:female ratio in the enriched egg group. In a follow-up clinical trial, males and females would be randomly allocated to the two treatment groups, and food colouring could also be added to both trial supplements to eliminate the need for different locations for both arms of the trial. Of note, previous work has shown that females responded better to carotenoid supplementation than males^(^ ^83^ ^)^. In addition, it has been reported that carotenoids in an egg matrix may possibly have significantly lower bioaccessibility, because of reduced retention and transfer of the carotenoids to the micelles (micellarisation), when cooked by scrambling (the method chosen in the current study) in comparison with boiling^(^ ^92^ ^)^.

In conclusion, we have shown that consumption of both normal and L- and MZ-enriched eggs significantly increased serum concentrations of MP’s constituent carotenoids after 8 weeks’ supplementation. Although measures of MP and the majority of measures of visual performance did not improve significantly in either study group, and given the observed significant increases in serum concentrations of MP’s constituent carotenoids, we feel that a study of greater duration is required before definitive conclusions can be drawn on the potential of carotenoid-enriched eggs to augment MP and/or impact favourably on vision. The finding that CisZ appeared to have greater bioaccessibility to serum than *trans* Z and MZ is potentially interesting, and warrants further investigation. In summary, carotenoid-enriched eggs could represent a cost-effective and readily bioaccessible source of the macular carotenoids as an alternative to over-the-counter formulations.

## Supplementary material

For supplementary material accompanying this paper visit http://dx.doi.org/10.1017/S0007114516003895. click here to view supplementary material
