# Supplementary material for: Serum and macular response to carotenoid-enriched egg supplementation in human subjects: the Egg Xanthophyll Intervention clinical Trial (EXIT)
Source: Br J Nutr. 2017 Jan 14;117(1):108–23. doi: 10.1017/S0007114516003895 (PMC5297582; doi:10.1017/S0007114516003895)
Supplement: Supplementary file 1 [file S0007114516003895sup001.doc]

**
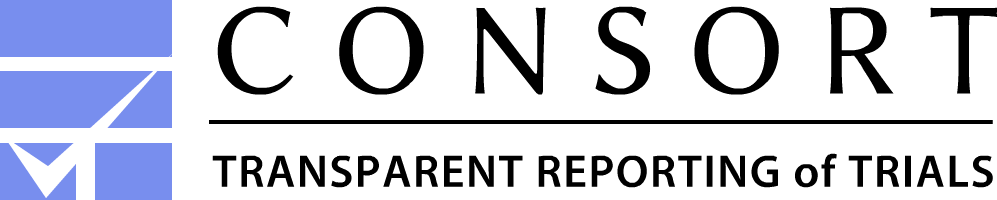
**

**CONSORT 2016 Flow Diagram for the Egg Xanthophyll Intervention Trial (EXIT)**

**Allocation**

**Analysis**

**Follow-Up**

**Enrollment**

Assessed for eligibility ( n = 52 )

Excluded ( n = 2 )

♦  Not meeting inclusion criteria ( n = 2 )

♦  Declined to participate ( n = 0 )

♦  Other reasons ( n = 0 )

Analysed ( n = 23 )
♦ Excluded from analysis ( n = 0 )

Lost to follow-up (n=2): Cholesterol level exceeded upper limit at 4 weeks ( n = 1 ); Personal reasons ( n = 1 )

Discontinued intervention ( n = 2 ); As above

Allocated to intervention ( n = 25 )

♦ Received allocated intervention ( n = 25 )

Standard control (placebo) egg

♦ Did not receive allocated intervention ( n = 0 )

Lost to follow-up (n=2): Cholesterol level exceeded upper limit at 4 weeks ( n = 1 ); Personal reasons ( n = 1 )

Discontinued intervention ( n = 2 ); As above

Allocated to intervention ( n = 25 )

♦ Received allocated intervention ( n = 25 )

Macular carotenoid enriched egg (active intervention), containing L:MZ in a 1:1 ratio

♦ Did not receive allocated intervention ( n = 0 )

Analysed ( n = 23 )
♦ Excluded from analysis ( n = 0 )

Randomized ( n = 0 )
